# Primary health care as a tool to promote equity and sustainability; a review of Latin American and Caribbean literature

**DOI:** 10.1186/s12939-024-02149-9

**Published:** 2024-05-06

**Authors:** Daniel Maceira, Rolando Enrique Peñaloza Quintero, Patricia Suarez, Laura Vanessa Peña Peña

**Affiliations:** 1 Economics Department, Universidad de Buenos Aires; CONICET/CEDES; Universidad de San Andrés, Health Systems Global, Buenos Aires, Argentina; 2 Institute of Public Health, Pontificia Universidad Javeriana, Bogotá, Colombia; 3 Center for the Study of State and Society (CEDES), Buenos Aires, Argentina

**Keywords:** Primary care, Health, Latin America, Caribbean, Healthcare networks, Health networks, Health care lines

## Abstract

Primary health care (PHC) has increased in global relevance as it has been demonstrated to be a useful strategy to promote community access to health services. Multilateral organizations and national governments have reached a consensus regarding the basic principles of PHC, but the application of these varies from country to country due to the particularities of local health systems.

This article aims to review and summarize PHC strategies and the configuration of health networks in Latin American and Caribbean countries.

The review was carried out using keywords in at least 9 databases. Papers in languages other than English, Portuguese, and Spanish were excluded, while non-refereed articles and regional gray literature were incorporated. As a result, 1,146 papers were identified. After three instances of analysis, 142 articles were selected for this investigation. Data were analyzed according to an analysis by theme.

The evidence collected on health reforms in the region reflects the need to intensify care strategies supported by PHC and care networks. These must be resilient to changes in the population’s needs and must be able to adapt to contexts of epidemiological accumulation.

**Supplementary Information:**

The online version contains supplementary material available at 10.1186/s12939-024-02149-9.

## Executive summary

The global relevance of primary health care (PHC) has increased as it has been demonstrated to be a useful strategy to promote community access to health services. Multilateral organizations and national governments have reached a consensus regarding the basic principles of PHC, but the application of these varies from country to country due to the particularities of local health systems.

The motivation for this article stems from the investigation of PHC models and strategies implemented in the region of the Americas and the observance of the configuration of health networks from a PHC perspective.

A systematic literature review was conducted using keywords in at least 9 databases. Languages other than English, Portuguese and Spanish were established as exclusion criteria, and non-refereed articles and regional gray literature were incorporated. As a result, 1,146 articles were identified. After three instances of analysis, 142 were selected. These were classified according to six thematic areas.

The evidence collected on health reforms in the region reflects the need to intensify care strategies supported by PHC and by consistent care networks that are resilient to changes in the population’s needs and that adapt to the contexts of epidemiological accumulation in the medium and long term.

## Introduction

Primary health care (PHC) is considered a key strategy in the area of health since it favors social development, promotes community participation and generates wellbeing for societies [22].

The concept emerged in 1978 within the framework of the International Conference held in Alma-Ata as a proposal to remedy a common need of all governments to maintain and guarantee their populations’ health, understood as *“a state of complete physical, mental and social well-being and not merely the absence of disease and infirmity”* [43, 123]. It considers health to be a fundamental right, incorporating practices of disease prevention and health promotion, and requires intersectoral coordination and the involvement of communities since the first level of care is the fundamental space for contact between populations and the health system [44].

Towards the end of the eighties, the WHO/PAHO demonstrated the need to organize health services into levels of care and to develop reference and counter-reference systems as an instrument for the regional planning of health services [46, 124]. The need for integrated and decentralized levels of care was ratified by PAHO Member States in 2005 through the Montevideo Declaration [48] and the Health Agenda of the Americas 2008–2017 [49]. The document “Integrated Health Services Networks” belonging to the series “The Renewal of Primary Health Care in the Americas” [51] constitutes the basis of health system reforms that, under the PHC paradigm, reorient, strengthen and deepen four major areas: the care model, network governance and strategy, the organization and management of resources in general terms and allocation and economic incentives.

Latin America’s countries have historically shown wide inequalities, not only in health outcomes, but also in income distribution, education, access to clean water and sanitation, among others [84]. At the beginning of the nineties, almost all Latin American and Caribbean countries had initiated, or were considering initiating, health sector reforms [54]. Several key policies were included as part of the menu: separation of functions, decentralization, improvement of health insurance schemes through nominalization of patients, and explicit package of services, among others. In some cases, they involved a wider reform of the State, including National Constitutions [14, 17, 20, 26, 28, 36, 37]. Different studies have shown that health system reforms in Latin American countries have promoted inclusion, citizen empowerment, and health equity, achieving in many cases the establishment of legal rights associated with health, and universal coverage [3, 82] even reducing health indicator gaps among income groups [34]. With the motivation of social justice and the achievements of equity, civil society played a leading role in obtaining citizen's rights and the right to health. An example of these has been the efforts made by the Latin American Social Medicine (LASM) [72]. At the international level, several organizations have had an important influence in the region in order to improve the health system's functioning and its results, as well as promoting the adoption of PHC proposal. Some of them are PAHO, WHO, UNICEF, and the World Bank.

With the arrival of the new century, PHC gained increased relevance at a global level [44] as a mechanism that provides effective responses to the needs of health systems. Among the different forms that the implementation of PHC has taken are the comprehensive (CPHC), selective (SPHC) and renewed (RPHC) approaches. The first, interpreted as part of an integrated system of health care and the economic-social development of a society that involves cooperation with other sectors [44], has been criticized for being too idealistic and lacking in implementability, making its application difficult in different communities [12]. Subsequently, discussions began on SPHC, which focuses on specific interventions aimed at the highest risk populations without incorporating the social component of equity, intersectorality and community participation characteristic of CPHC [74]. Finally, RPHC emerged as an approach that combines a family and community perspective aimed at strengthening current health systems, seeking equity and sustainability in access and the provision of health services with defined populations and territories. In this way, it carries elements of citizenship, with social participation and emancipation [50, 125, 126].

During the eighties and nineties, in some countries SPHC models were adopted through programs aimed at specific population groups [29], and later, at the beginning of the twenty-first century, other countries came to the adoption of RPHC, promoted by international organizations, to achieve systems that guarantee universal access to health services [47]. Nevertheless, the interpretation of the scope of one PHC model or another is highly disputed in Latin America, and the heterogeneity in the way they are implemented reflect the political and economic instability of the region [9, 62, 67, 146, 147].

Analyzing different experiences of implementing PHC models, as well as the development and configuration of health care networks, can contribute to a better understanding of the scope of these tools, and their contribution to promoting equity and sustainability of health care systems. In this sense, this review seeks to demonstrate the ways in which the countries of the region have approached primary health care, paying special attention to two components: the experiences of implementing PHC models or strategies and the treatment of health networks. To these ends, an integrative review^1^ was carried out during the period of December 2022 and May 2023, in order to systematically map the research conducted in this field based on the following research questions: how have PHC models and strategies been implemented? How have health networks been configured under a PHC perspective?

Articles published between 1982–2022 with qualitative, quantitative and mixed methodologies were included. The definition of the temporal space considered in the study reflects three different periods experienced by Latin American health systems. During the eighties, health systems mainly involved a supply subsidy approach, which shaped the development of traditional health provision structures. Subsequently, during the nineties, health systems reforms were embedded by a demand-side approach based on payment incentives. With the new century, the limitations of both approaches became visible, emerging mixed models as part of the menu for sustainable reforms.

**Fig. 1:**
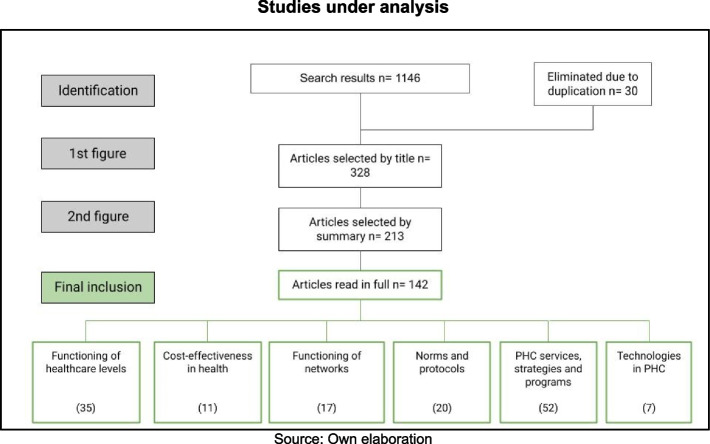
Studies under analysis. Source: own elaboration

The review identified six priority areas: the functioning of healthcare levels; cost-effectiveness in health; the operation and experiences of health networks; regulations and protocols used in health systems; services, strategies and programs oriented towards PHC; and new technologies implemented for PHC (Fig. 1)

## The Latin American experience in PHC and health networks

In Latin America, public systems for the provision of health services have relied on PHC to address common problems such as the fragmentation of systems; the decentralization of care; the scarcity of economic and professional resources; and the indiscriminate demand related to high- and low-complexity pathologies in specialized centers [97, 100–102, 104, 106, 108, 109, 129, 134, 135, 138, 140].

Some countries have strengthened the logic of networks and levels of care [96, 114, 115]. In such cases, under the PHC model, the offer is organized from the first level of care to the third or fourth level of complexity and, among other characteristics, provides for universal services. On the supply-side, the objective is to break down economic barriers by establishing user fees that are usually zero or relatively low (compared to those of the private system). Meanwhile, each component of the network is responsible for a defined population, whose members must be identified and assigned responsibilities. The assignment of responsibilities must follow a risk criteria for the beneficiaries so as to efficiently cover different needs. In this way, each country will develop the network that is most appropriate according to the epidemiological, demographic, sociocultural and economic characteristics of its population, that is, considering not only the possibilities offered by the reform of supply but also the characteristics of demand [33, 110, 128].

The selection of nations^2^ seeks sub-regional representation, involving Mexico, Brazil, Central American countries, Andean countries, and the Southern Cone, providing special attention to differentiated health care organizations. Costa Rica, Chile, Brazil and Colombia were included as paradigmatic cases of PHC models, while Bolivia and Guatemala were included as nations with the presence of indigenous populations, needs and health perceptions, while Argentina and Uruguay represent countries with relatively high levels of socioeconomic development and recent programmatic reforms. In alphabetical order, below are the experiences of the countries mentioned.

In Argentina, Plan Nacer, which was designed in 2004, initially focused on the population of mothers and children that lacked formal insurance (with exclusive public coverage) in nine priority provinces in the north of the country. It was implemented based on the registration of users and the establishment of payments tied to the fulfillment of management commitments agreed between the National Ministry of Health and its provincial counterparts [111, 112, 122, 148]. The initiative was expanded to the rest of the country and to all social groups, adding coverage for specific lines of care [83, 116]. This format is maintained to the present day. In parallel, the Networks Program, Community Doctors Program (PMC) and the Remediar program were implemented.^3^ The first of these proposed the promotion of equitable access to health services for those segments of the population without coverage; the second financed human resources and training at the first level of care; the third focused on providing coverage of essential medicines through primary care centers [51, 39, 64, 117, 118]. Based on interviews with key actors, [66] recorded that the financing of the PMC was limited and did not guarantee the insertion of professionals in the system. Meanwhile, Maceira, D., et al. [31], – through surveys of the user population and a control group and comparison of program purchase prices and secondary sources – and Tobar, F., [68] – through secondary descriptive statistics – showed that the Remediar Program allowed efficient purchases with a redistributive impact due to its focus on the PHC network, reducing out-of-pocket expenses for lower-income families.

In Brazil, the Family Health Strategy (ESF) has configured a person-centered PHC model. Its predecessor, the Health and Family Program, initiated in the 1990s, promoted the continuous training of health professionals and managed to improve indicators of malnutrition, infant mortality and hospitalization due to dehydration, among others [8]. In fact, a study carried out in 2006 [35] showed – through an econometric analysis – the association between program coverage and lower infant mortality rates, reflecting the program’s contribution to the fall in child mortality. Puentes Vacca, J. S., and Torres Ruda, Y. M. [58] point out that the ESF strengthened links between the community and actors in the health system as well as local and regional empowerment. However, Carneiro Junior, N., and Gené Badia, J., [7] criticize the ESF’s rigidity, noting that it does not take account of different regional realities, although they highlight the presence of greater annual resources allocated to PHC, a fundamental element for the consolidation of the Brazilian National Health System [50]. Regarding care networks, various studies have shown that their implementation has positively influenced the survival of people with breast cancer [13, 55, 98]. However, experiences of informality and an appropriation of resources in some municipalities show the need for greater levels of auditing and control [24, 56, 113, 130, 136, 137, 144].

In Bolivia, a series of reforms^4^ developed during the nineties gave rise to the Intercultural Community Family Health Model (SAFCI), which functions as a PHC strategy, contemplating the principles of community participation, intersectorality, interculturality and integrality [21]. Although research on the current Bolivian health system and the operation of the SAFCI model is limited [2], during the last decades, Bolivia has shown improvements in the general implementation of the health system, promoting social participation in decision-making linked to health management and generating programs to approach the health of native peoples [57], which has improved indicators of prenatal care and the treatment of tuberculosis, etc. [45]. At the same time, the country has made progress in the formation of health networks in order to reduce the levels of maternal and infant mortality. The Integrated Health Service Delivery Networks (IHSDN) model is operationalized in the networks of municipal and departmental health services that must connect with the third level. The literature has shown that although efforts have been made to give each municipal network a reference center at the higher level, there remain difficulties in achieving adequate coordination [67].

The reforms that emerged in the year 2000 in Chile enabled the subsequent implementation of the country’s PHC strategy, giving rise to the creation of public organizations focused on health management and the development of service networks [19]. The Universal Access and Explicit Guarantees Plan (AUGE), in particular, is responsible for providing protection for certain priority pathologies [53, 119]. A study analyzed the situation before and after the implementation of this plan for patients with chronic kidney failure and determined that the main challenges were associated with a lack of human resources and equipment [25]. In contrast, another study evaluated the results in patients with acute myocardial infarction using econometric techniques and showed an association between the implementation of the AUGE Plan and a reduction in in-hospital mortality in at-risk patients undergoing thrombolysis and aged over 75 years, where other strategies were yet to achieve a great impact [42].

In the case of Colombia, a PHC strategy was initiated in Bogotá starting in 2004 based on the Salud a su Hogar Program (SHP), which aimed to reduce social inequality, improve access and use of health services and to promote the participation of civil society and communities [80, 95, 121]. A study carried out in 2008 based on an analysis of official data showed that implementation of the strategy had helped to improve the indicators of infant mortality, post-neonatal mortality and mortality due to pneumonia in children under 5 years of age [40]. Another study, using descriptive statistics, concluded that although the primary care model has been linked to an improved performance of health services, the aspects of access, family focus and community orientation are the least developed [60].

In Costa Rica, PHC reform was based on the establishment of multidisciplinary teams, community participation and the vertical integration of care at all levels. What stands out here are the measures of coverage and use of health services, whose performance has been associated with the population’s state of health and high life expectancy [69]. A quasi-experimental study attributes the decrease in infant and adult mortality to the reforms in PHC. In particular, it points out that the programs within the framework of the PHC strategy led the infant mortality rate to fall by 8%, the adult mortality rate by 2%, and inequity in access from 30 to 22% between 1985 and 2001 [61]. On the other hand, a study on the efficiency of primary health care spending identified a need to adjust the scarce resources of the Costa Rican Social Security Fund (CCSS) to respond to the current demographic change [27].

The experience of El Salvador with its Basic Comprehensive Health Systems (SIBASIS) has promoted access via the IHSDN model, promoting comprehensive care through Community Family Health Teams (ECOS). Here, the coordination of health networks based on the involvement of families and the community has led to improvements in the elimination of geographical, economic and cultural barriers affecting the Salvadoran population’s access to health services [5, 90].

In Guatemala, the Health Coverage Extension Program (PEC), with an emphasis on mother and child care, seeks to counteract a limited state offer in a country where 57% of the population still resorts to private services (PAHO, 2010). In this case, care networks not only seek to achieve efficiency in the allocation of resources but also to confront the challenge of improving access and expanding the coverage of services.

In Mexico, asymmetries have been observed between care provided by the health system and the needs of the population because an unequal geographical distribution of infrastructure reproduces regional inequalities, coinciding with the geographical distribution of poverty [71, 127, 131]. A recent study analyzing the barriers to access health services showed that, in 2018, 30.9% of the country's localities did not have physical access to basic health services. Among the main barriers are the travel time to health facilities, averaging 53.4 min [10]. The reforms produced by the General Health Law of 2003 gave rise to the Seguro Popular (SP), which sought to provide coverage to those who lacked social security [139]. Although some reports have indicated that it has helped to increase access for the most vulnerable sectors, Durán-Arenas, L., et al. [16] observed that those covered by social security and the popular insurance program do not receive the same type of coverage. They highlight a lack of indicators to allow an observation of the SP’s performance. At the same time, another study recorded an insufficient number of professionals to carry out health promotion and disease prevention activities and highlighted a need to adapt profiles to the established tasks [1]. A reform has recently been implemented, through which the SP has been replaced by a new organization: the Health Institute for Social Welfare, although the literature has not yet been able to analyze its performance.

In Paraguay, PHC has been developed through vertical programs with little connection or coordination. Although Law 1032 of 1996 recognized the importance of community participation in the implementation of health policies, plans and programs (aligned with the characteristics of PHC), 40% of Paraguayans do not have access to health services [58]. Among the main weaknesses, a lack of human and financial resources has been observed, along with the weak governance of public organizations [15]. Furthermore, through interviews with key actors, Villalba, B., et al. [73] identified a need to increase the level of qualification among professionals.

For its part, Uruguay created the National Integrated Health System (SNIS) in 2007. This established the organization of networks by level of care, prioritizing the first level of care under a PHC approach [21, 58, 120]. Sollazzo, A., & Berterretche, R., [65] observed that the segmentation of the system and the low level of coordination between levels of care negatively affected general health indicators, and that the lack of human resources constituted a threat to the PHC strategy.

## Priority areas for the development of PHC strategies and health networks

While access and continuity in health care are two essential objectives in any primary care strategy [41], the presence of a high level of unmet health needs in populations dependent on both public and private sectors’ coverage demonstrates that care requirements exceed the capacity of resolution of a single provider [81, 91, 92]. Health outcomes -and outputs- are the result of a collaborative job, where different levels of care and multidisciplinary cooperation combined are required [133]. Therefore, how to implement effective health care networks, and how to guarantee the provision of care in segmented systems though a sound regulatory framework are part of a comprehensive PHC strategy.

The presence of healthcare networks operationally dependent on several intersecting jurisdictions -product of decentralized and/or segmented systems- makes the necessary coordination even more complex [32, 132, 142].

Factors commonly identified as obstacles to efficiency in networked healthcare system are the failure in the referral and counter-referral mechanisms; the absence of standardized information about patients; a failure in the performance of the body responsible for coordinating the service network; the lack of commitment and/or instruction of health personnel; and the absence of a perspective of ethnic and cultural diversity [11, 30, 38, 49, 59, 63, 76, 77, 85, 86, 89, 94, 103, 107]. As such, the discussion about the reasons why a health care network fails requires a comprehensive approach [23, 87].

Key elements that contribute to a better functioning of health care levels are financial incentives and the mechanisms of technology incorporation. The first -together with a wide range of policies oriented to strengthening professional careers- could stimulate healthcare personnel to switch clinical behavior towards preventive, sound diagnostic and informed treatment decisions [18, 79]. Reputation incentives contribute in the same way, particularly when information on the performance of professionals is published [6]. In turn, the incorporation of technologies has proven to be useful to facilitate learning, cooperation, better promotion and monitoring procedures, especially in rural setups, constituting a complement to care at the first level [52, 70, 75, 88, 93, 99, 141, 143].

Beyond this, the presence of different jurisdictions represented within a network opens a discussion on the structure of the vertical integration of services [145], not only in ownership but also in the design of spaces of governance, control and coordination [78]. Where budgetary sources are not necessarily aligned, the integration of services should shift to the presence of regulatory and protocolized ties that allow the distribution of tasks and responsibilities, achieving complementarity, which requires a common monitoring structure [32, 105]. Network governance is thus a key aspect that managers and designers must take into account when establishing priorities as part of a PHC plan. In brief, the leadership to unite wills and modify deep-rooted processes is the starting point for network governance [33].

Therefore, the institutional design of the region's health systems, political and economic disputes and the values ​​of the different actors involved are key elements that determine the regulation of PHC [4]. As such, the disconnect among actors and the low leadership capacity in health systems condition the design of such strategies.

## Conclusions

Although we recognize achievements in the process of building PHC and health networks in Latin America, which results are reflected in key health indicators, there are still key issues to be addressed based on the evidence analyzed.

First of all, the concept of primary health care is clearly rooted in institutional discourse and in the sectoral debate in the countries of the region. However, the approach is challenged by the organizational structure of the health systems themselves. Intense fragmentation, ineffective decentralization and the accumulation of programs along the line of care lead to the consolidation of a care model that deviates from what is considered good practice.

Profound inequality in income distribution operates as a segmentation mechanism in itself, generating different levels of coverage associated with the ability to pay. During the last decades, efforts have been observed in almost all countries in the region to promote coordinated models of care, although their results have been uneven and monitoring and evaluation of the impact achieved is scant.

The little coordination between subsystems amplifies differences and leads to the development of disjointed health networks strongly based on informality and a lack of protocolization. Dialogue between providers by subsystem is sparse, and the development of the private sector is not seen to be a space for coordination and complementation with the public and -in some cases- social security system but rather a mechanism that deepens care gaps.

The evidence collected for this review has highlighted some key themes addressed throughout the document. Among them, the need to intensify PHC models and care networks stands out, following the results documented. At the same time, the document identified the lack of coverage of other relevant topics, such as how health systems adapt to changes in population needs, and the adaptation to health systems to contexts of epidemiological accumulation, including mental health, addictions and environmental impacts.

In addition, this review found limited literature documenting changes of financial and non-financial incentives and their impact on health services’ provision, efficiency in the allocation of resources, as well as quality improvements, opening the room for future lines of research. This result might show the need for deeper interaction between research and political action in the Latin American and Caribbean region to facilitate an exchange of information, strengthen impact evaluation of interventions, and jointly design a research-action agenda with social impact.

### Supplementary Information

**Additional file 1. Methodology.**

## Data Availability

No datasets were generated or analysed during the current study.

## Abbreviations

AUGE: Universal Access and Explicit Guarantees Plan
CCSS: Costa Rican Social Security Fund
CPHC: Comprehensive Primary Health Care
ECOS: Community Family Health Teams
ESF: Family Health Strategy
IHSDN: The Integrated Health Service Delivery Networks
LASM: Latin American Social Medicine
PEC: Health Coverage Extension Program
PMC: Community Doctors Program
PHC: Primary Health Care
RPHC: Renewed Primary Health Care
SAFCI: Intercultural Community Family Health Model
SHP: Salud a su Hogar Program
SIBASIS: Basic Comprehensive Health Systems
SNIS: National Integrated Health System
SP: Seguro Popular
SPHC: Selective Primary Health Care

## Footnotes

1 A more complete methodological justification is included in the supplementary material section.

2 The structure used to describe what is being done in each country has been defined by the authors. Even though we recognize the value of the evaluation’s schemes for health systems programs and initiatives used by international organizations such as WHO, UNICEF, etc. we considered this way fits the objectives of the paper and allows us to better organize the evidence found.

3 Launched under Ministerial Resolution 844/2022, the current National Community Health Program proposes to continue the objective of strengthening the national PHC strategy.

4 Popular Participation Law (Law No. 1551), Administrative Decentralization Law (Law No. 1654).
