# Supplementary material for: Primary health care as a tool to promote equity and sustainability; a review of Latin American and Caribbean literature
Source: Int J Equity Health. 2024 May 6;23:91. doi: 10.1186/s12939-024-02149-9 (PMC11075272; doi:10.1186/s12939-024-02149-9)
Supplement: Supplementary file 1 — Additional file 1. Methodology. [file 12939_2024_2149_MOESM1_ESM.docx]

**Supplementary material**

**1.- Methodology**

An integrative review was carried out during the period of December 2022 and May 2023, in order to systematically map the research conducted in this field based on the following research questions: how have PHC models been implemented? How have health networks been configured under a PHC model? To this end, articles published between 1982-2022 with qualitative, quantitative and mixed methodologies were included.

The definition of the temporal space considered in the study reflects three different periods experienced by Latin American health systems. During the eighties, health systems mainly involved a supply subsidy approach, which shaped the development of traditional health provision structures. Subsequently, during the nineties, health systems reforms were embedded by a demand-side approach based on payment incentives. With the new century, the limitations of both approaches became visible, emerging mixed models as part of the menu for sustainable reforms.

The search was carried out in the databases: SciELO, LILACS, ScienceDirect, Scopus, Doyma, Pubmed, Proquest, Medline EbscoHost, Wiley Online Library. Three search equations were used: ("Primary Health Care" OR PHC OR "Primary Care") AND ("health system" OR "health systems") AND ("Latin America" ​​OR "Latin America" ​​OR Caribbean); as well as ("Primary Health Care" OR PHC OR "Primary Care") AND ("health system" OR "health systems") AND ("Latin America" ​​OR "Latin America" ​​OR Caribbean) AND cost; and (“Health Networks” OR “Health Networks” OR “Care Lines” OR “Health Care Lines”).

Languages ​​other than English, Portuguese and Spanish were established as exclusion criteria. Non-refereed working papers, publications and regional gray literature were included -produced by leader health multilaterals such as PAHO, WHO, as well as government institutions, universities and regional research centers, based on the review provided by the Latin American Collaboration for Research in Health Policies and Systems (CLIPSS).

Empirical studies and/or applications and implementations of primary care models were included. Management experiences, program evaluations based on qualitative, quantitative and/or mixed methodologies, as well as case studies were considered. Essays and opinion articles were excluded. Based on these inclusion and exclusion criteria, each article was analyzed by title and abstract. Finally, the selected articles were read in full and a final purification of the results was undertaken.

Six priority themes were identified due to their recurrence in the searches conducted: the functioning of healthcare levels; cost-effectiveness in health; the operation and experiences of health networks; regulations and protocols used in health systems; services, strategies and programs oriented towards PHC; and new technologies implemented for PHC. The texts were systematized in an analysis matrix according to the criteria presented in Figure 1.

Of the studies included (n= 142), 111 were undertaken in Latin America. Of the total number of works, 35 address the functioning of healthcare levels, among which econometric analysis and descriptive statistics predominated along with essays, to a lesser extent. The cost-effectiveness studies (n= 11) presented evaluative objectives and used descriptive quantitative methodologies and, to a lesser extent, econometric analysis. The field on the functioning of health networks (n= 17) presented descriptive works that combined interviews with key actors and analysis of data sets. Although normative texts were consolidated with those focused on protocols (n= 20), the former were characterized by being descriptive, while the latter mostly used qualitative methodologies oriented towards both health professionals and recipients. The works on technologies applied to PHC (n= 7) were concerned with describing the devices used under a case study methodology. Finally, the articles that addressed PHC services, strategies and programs (n= 52) were predominantly essays oriented towards descriptions of PHC programs, mechanisms and configuration, access to services and knowledge about the system, challenges and opportunities, and proposals for reforms.
